# Tiger Habitat Occupancy in Chitwan–Parsa Complex: Implications for Human–Tiger Conflict Management Strategies

**DOI:** 10.1002/ece3.72739

**Published:** 2025-12-18

**Authors:** Anil Prasai, Saneer Lamichhane, Abhinaya Pathak, Ajay Karki, Trishna Rayamajhi, Jeffrey Mintz, Bhagawan Raj Dahal, Bed Kumar Dhakal, Chiranjibi Prasad Pokheral, Naresh Subedi, Bishnu Prasad Bhattarai

**Affiliations:** ^1^ Central Department of Zoology Tribhuvan University Kathmandu Nepal; ^2^ National Trust for Nature Conservation Lalitpur Nepal; ^3^ School of Natural Resources and Environment University of Florida Gainesville Florida USA; ^4^ Department of National Parks and Wildlife Conservation Kathmandu Nepal; ^5^ School of Biological Sciences University of California San Diego California USA; ^6^ Haub School of Environment and Natural Resources University of Wyoming Laramie USA; ^7^ Department of Earth and Environment Florida International University Miami Florida USA; ^8^ Zoological Society of London Kathmandu Nepal

**Keywords:** Chitwan–Parsa complex, habitat use, human–tiger conflict, occupancy, tiger, tiger rescue

## Abstract

The rebound of tiger populations in Nepal over the last decade has renewed hope for species conservation but also heightened the risk of conflict where humans and tigers coexist. Most of these tigers inhabit Chitwan–Parsa Complex (CPC), which includes core areas prohibiting humans and buffer zones allowing limited activities. To understand distribution within CPC and nearby forests, we constructed a Bayesian occupancy model using data from a sign survey conducted between December 2021 and February 2022. We estimated occupancy for 2021–22 dry‐season within CPC on a 10 km × 10 km gird and as well as the use by tigers and prey on a 2 km × 2 km subgrid. The average estimated occupancy ψ within 10 km × 10 km grids and detection p within 2 km × 2 km subgrids were 0.90 (95% CI 0.77–0.99) and 0.34 (95% CI 0.32–0.36), respectively. The presence of tigers was more strongly related to prey occupancy at the home‐range scale whereas factors such as vegetation, human population density (HPD), and elevation affected used portion of home range. HPD significantly reduced habitat use by tigers and prey. We compare our modeled tiger‐use distribution to an independent dataset containing conflict causing tiger rescue and relocation records. Tigers use only increased odds of rescues occurring in a subgrid by 10%, but subgrids with above average HPD had 2.2 times higher odds of rescues than those with low HPD, and the grids with above average prey use had 3.8 times higher odds of rescues than those with low prey. The pattern of increased rescues in high‐prey‐use areas was driven by subgrids with above average HPD, where the odds of rescues were 10.98 times higher than those with low prey use. The varying odds of conflict by HPD and prey use suggest future approaches to tiger conservation and conflict resolution.

## Graphical abstract

The rebound of tiger populations in Nepal over the last decade has renewed hope for species conservation but also heightened the risk of conflict where humans and tigers coexist. Most of these tigers inhabit Chitwan–Parsa Complex (CPC), which includes core areas prohibiting humans and buffer zones allowing limited activities. Tigers were found to be more strongly related to prey occupancy at the home‐range scale, whereas factors such as vegetation, human population density, and elevation affected which portion of the home range was used. Human population density significantly reduced habitat use by tigers and prey. We compare our results against “tiger rescue” records, where veterinary teams track and relocate specific tigers involved in conflicts. We found that in the buffer region, 2.8 times more rescues occurred in subgrids with above‐average estimated use. The high number of tiger rescue cases within high‐use buffer regions indicates an increased likelihood of human–tiger conflict in those areas and could inform future approaches to tiger conservation and conflict resolution.

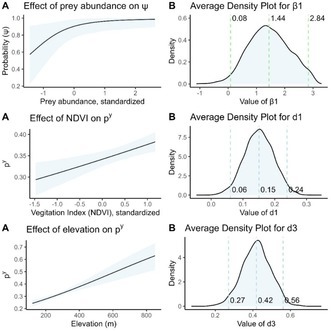

## 1 Introduction

The tiger ( *Panthera tigris* ) stands as the largest and most charismatic feline species globally (Kitchener et al. 2017). Over the centuries, they have symbolized a significant cultural icon, and served as an umbrella species, signifying the overall health of the ecosystem in Asia (Karanth 2003; Sunquist 1999). Yet, the expansion of the human population has triggered habitat degradation, causing a decline in the abundance and distribution of their prey. Moreover, hunting for skins and bones, along with agricultural expansion, has substantially reduced their number and fragmented the population and adversely impacted the population dynamics (Sunquist 1999; Karanth and Madhusudan 2002; Reddy et al. 2004). Approximately 93% of their historic range has been lost, resulting in their population plummeting from 100,000 to ~4500 within a century (Wikramanayake et al. 2010; Goodrich et al. 2022). Out of nine genetically distinct subspecies of tiger, only five—Amur, Bengal, Indochinese, Malayan, and Sumatran (extinct: Caspian, Javan, and Balinese)—survive today (Luo et al. 2008). Shockingly, the number of captive tigers exceeds the number of those inhabiting the wild (Nowell and Jackson 1996; CNN 2021).

Of the remaining subspecies, the Royal Bengal Tiger ( *Panthera tigris tigris* (Linnaeus 1758), referred to as ‘tiger’ hereafter), is indigenous to the Indian subcontinent and is found in Nepal, India, Bangladesh, and Bhutan (Kitchener et al. 2017; Sunquist and Sunquist 2002). Until the 1950s in Nepal, the southern lowlands, which served as tiger habitats, were preserved from development under the Rana Regime to create a malaria barrier from possible enemy attacks from the south. However, following the conclusion of the British Colonial Empire in India and the Rana Regime in Nepal in the 1950s, accompanied by the burgeoning human population in Nepal's midhills and an extensive malaria eradication program in the southern lowland, there was an upsurge in human settlements (Smith et al. 1999). These developments led to habitat fragmentation, segregating tigers into three distinct populations‐ Chitwan, Bardia, and Shukla populations (Smith et al. 1999; Karki et al. 2011; DNPWC and DFSC 2018, 2022). To address these concerns, the Government of Nepal formulated the National Park and Wildlife Conservation Act 1973, establishing the Chitwan National Park in the same year (DNPWC 2023). In 1984, the adjoining area east of Chitwan National Park, spanning 499 km^2^, was declared a wildlife reserve known as the Parsa Wildlife Reserve (now Parsa National Park). Currently, these two protected areas collectively constitute the Chitwan–Parsa Complex (CPC), which is a part of a larger landscape that includes Valmiki National Park, India. Together they form the largest sub‐Himalayan tiger population. The forested area is not restricted solely to these protected areas but extends continuously further east and north of Parsa National Park and further west and north of Chitwan National Park, much of which is incorporated into a buffer system. The parks' buffer zones simultaneously promote conservation while enabling local people to use forest resources to enhance their economic circumstances (HMGN [His Majesty's Government of Nepal] 1996; DNPWC 2023).

In 2009, Nepal conducted its first comprehensive assessment of tigers at a national level, estimating the tiger population at 121 (95% CI 100, 191) individuals within four tiger bearing lowland protected areas—Parsa, Chitwan, Bardia, and Shuklaphanta. Among these, Chitwan National Park harbored the largest number with 91 (95% CI 71, 147) tigers, while the adjoining Parsa National Park accounted for four tigers (95% CI 4, 4) (Karki et al. 2011). In the same year, Nepal committed to doubling its tiger population by 2022 by signing the St. Petersburg agreement. Presently, the tiger population has notably risen in Chitwan and Parsa national parks, reaching 128 (95% CI 121, 140) and 41 (95% CI 38, 50) individuals, respectively, up to 355 (95% CI 334, 403) tigers across Nepal, effectively doubling the 2010 census (DNPWC and DFSC 2022). The Chitwan–Parsa Complex (CPC) boasts the highest number of tigers (*n* = 169) compared to other lowland protected areas. The 2022 report highlighted tiger sightings east and west of the CPC (DNPWC and DFSC 2022).

With rising tiger and human densities in the buffer zones of lowland protected areas such as Chitwan and Bardia National Parks, the frequency of human–tiger conflict has increased (Bhattarai et al. 2019). For example, in Chitwan National Park, from 2007 to 2014, an average of 4 people were killed, 2.7 injured, and 44 livestock lost annually (Dhungana et al. 2017). When such conflict incidents become frequent, protected areas take measures to capture these tigers (CNP 2022). For example, from 2007 to 2016, 22 tigers were captured in Chitwan National Park, including 13 that killed humans, six that targeted livestock, and three posing threats without casualties (Lamichhane et al. 2017).

The capture and relocation of injured, orphaned, or conflict tigers across Nepal are conducted by dedicated Wildlife Rescue Teams under the DNPWC with technical assistance from the National Trust for Nature Conservation field offices. These interventions, known as tiger rescues, are a multidisciplinary effort involving veterinarians, technicians, biologists, security personnel, official and local leaders. Rescue teams must coordinate permissions, secure sites and crowds, perform tiger identification (e.g., sex, possible age and weight) and chemical immobilization, deliver clinical stabilization, and ensure humane transport. Using species‐specific tools and protocols (e.g., vit‐cloth containment, remote drug delivery, PPE, and physiological monitoring), they transfer tigers to pre‐determined release sites or to rescue centres for further treatment under protected‐area authority. This rapid, coordinated capacity is now central to mitigating human–wildlife conflict while safeguarding animal welfare (DNPWC 2025).

Higher tiger occupancy in buffer zones and areas near human settlements likely contributes to increased conflicts and subsequent tiger rescues. Dhungana et al. (2017) found that 75.9% of human casualties from tiger attacks occurred in the buffer zone, with 66.7% within 1 km of the park boundary. However, whether conflict/rescue locations align with tiger occupancy patterns in the park's buffer zones remains unclear. To address this, we analyzed data on tiger rescues from July 2020 to September 2024 to assess whether there were any associations. This approach aims to determine whether human–tiger conflict is more prevalent in areas with higher tiger occupancy, offering valuable insights for managing conflicts and conserving tiger populations.

The objectives of our study are as follows: (i) to estimate tiger occupancy in the CPC and adjacent forests near human settlements during the 2021–22 dry season, and (ii) to assess the relationship between recent human–tiger conflict and tiger occupancy within the buffer zones. Concurrently, we proposed the following hypotheses: (i) Higher human population density would negatively affect tiger occupancy due to increased human disturbance. (ii) Normalize Difference Vegetation Index (NDVI) would positively influence tiger occupancy by providing better cover and hunting opportunities (Sharma et al. 2015). (iii) Terrain ruggedness would negatively impact tiger occupancy, as tigers generally avoid rugged landscapes (Lamichhane et al. 2025). (iv) Increased sampling effort (total km of search path per grid) enhances tiger detection, while dense vegetation and leaf litter (high NDVI) hinder it (Harihar and Pandav 2012).

By identifying conflict‐prone areas and understanding the spatial dynamics of tiger presence, our findings will provide crucial insights for implementing conflict mitigation measures, habitat management, and community engagement initiatives. This knowledge is essential for sustaining the growing tiger population while minimizing conflict risks, thereby contributing to long‐term conservation goals and effective management of tiger habitats in Nepal.

## 2 Methods

### 2.1 Study Area

**FIGURE 1:**
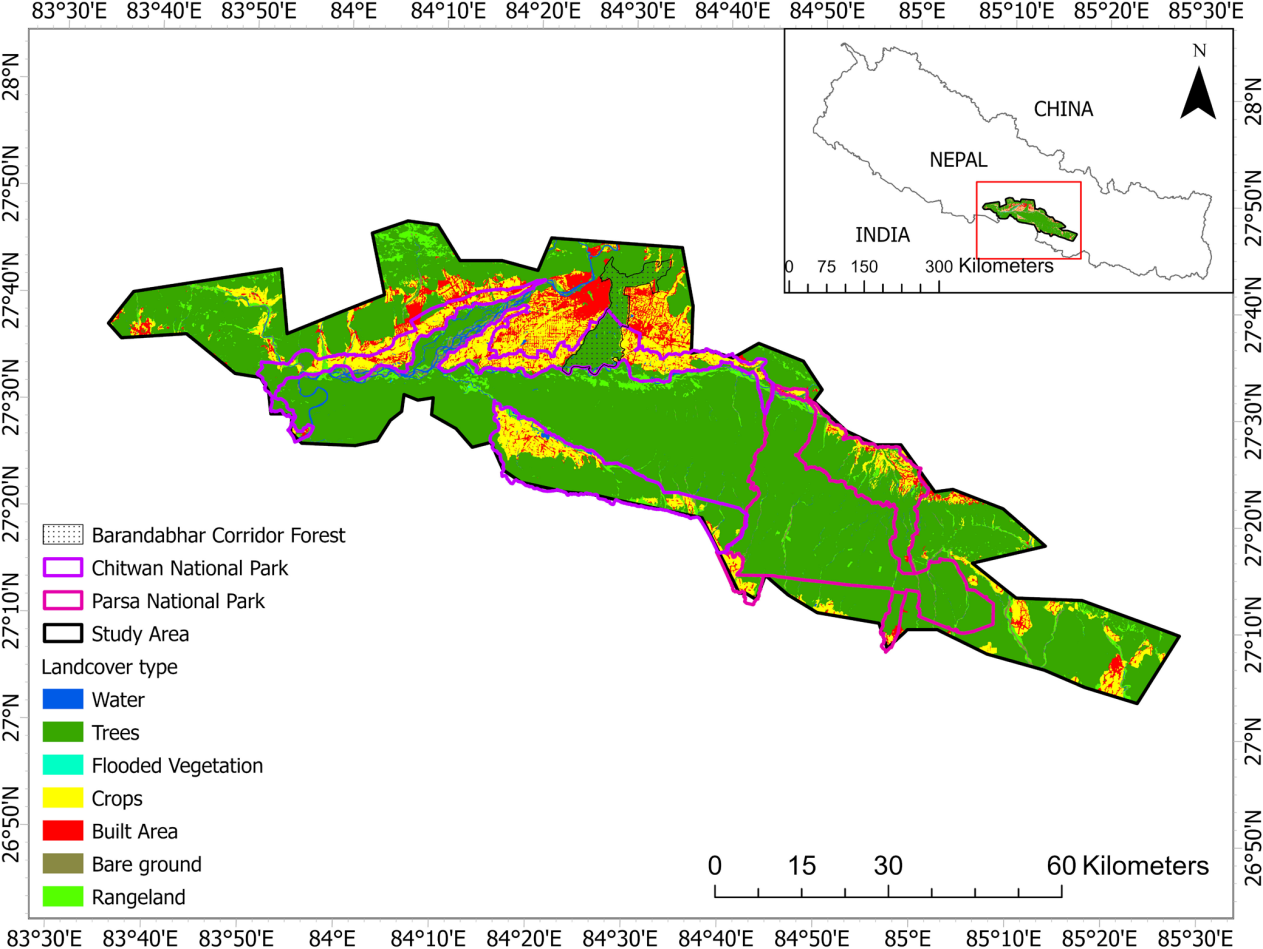
The study area covers both the Chitwan and Parsa national parks of the southern lowland of Nepal and extends the forested area further east, west, and south where the chances of tiger movement exist. “NP” and “BZ” refer to national park and buffer zone respectively.

Our study area spans 5700 km^2^ and situated in the south‐central lowlands of Nepal, primarily around the 2595 km^2^ Chitwan–Parsa complex (Figure 1). The complex encompasses Chitwan and Parsa National Parks and their buffer zones and extends into adjacent forested areas that facilitate tiger movement (DNPWC and DFSC 2022). The CPC shelters diverse wildlife including at least 68 mammal species, 544 bird species, 56 herpeto‐fauna, and 126 fish species (CNP 2023; PNP 2023; DNPWC 2023). Beyond tigers, the CPC supports sympatric carnivores including dholes ( *Cuon alpinus* ), leopards ( *Panthera pardus* ), and several smaller carnivore species like civets and mongoose (DNPWC 2023).

Chitwan National Park, the first national park in the country and a UNESCO World Heritage Site, boasts a diverse landscape. Bordered by the Valmiki Tiger Reserve in the south (India) and lesser Himalaya in the north, it is connected through the Barandabhar Corridor Forest and Daunne Forest, which extend to the Chure range in the west. The Chure, a young mountain range, comprises fragile sedimentary rocks covering 13% of the country (PCCP, n.d.). The dominant vegetation is the sal ( *Shorea robusta* ) forest, interspersed with chir pines (*Pinus roxburgii*) in the southern region. The riverine forests, constituting ~7% of the total area, exhibit rich plant diversity. Additionally, around 10% of the park is grassland, hosting species like elephant grass ( *Saccharum ravennae* ), giant cane ( *Arundo donax* ), and kans ( *Saccharum spontaneum* ). In addition to its population of tigers and leopards, this park is particularly renowned for its significant populations of the threatened Greater one‐horned rhinoceros (*Rhinocerous unicornis*) and the critically endangered gharial ( *Gavialis gangeticus* ) (CNP 2022, 2018).

Parsa National Park (PNP) was originally established as a wildlife reserve, which was upgraded to a national park in 2017. It shares its western border with Chitwan National Park and its southwestern boundary with Valmiki Tiger Reserve, India. The Rapti River and Siwalik Hills serve as natural boundaries, limiting human settlements to the north. The park's vegetation predominantly consists of tropical and moist deciduous vegetation where 90% of the vegetation is covered by sal forest. Tree species such as khair and sissoo thrive along the riverbanks, sometimes intermingling with chirpine (PNP 2023).

### 2.2 Study Design for Occupancy Survey

**FIGURE 2:**
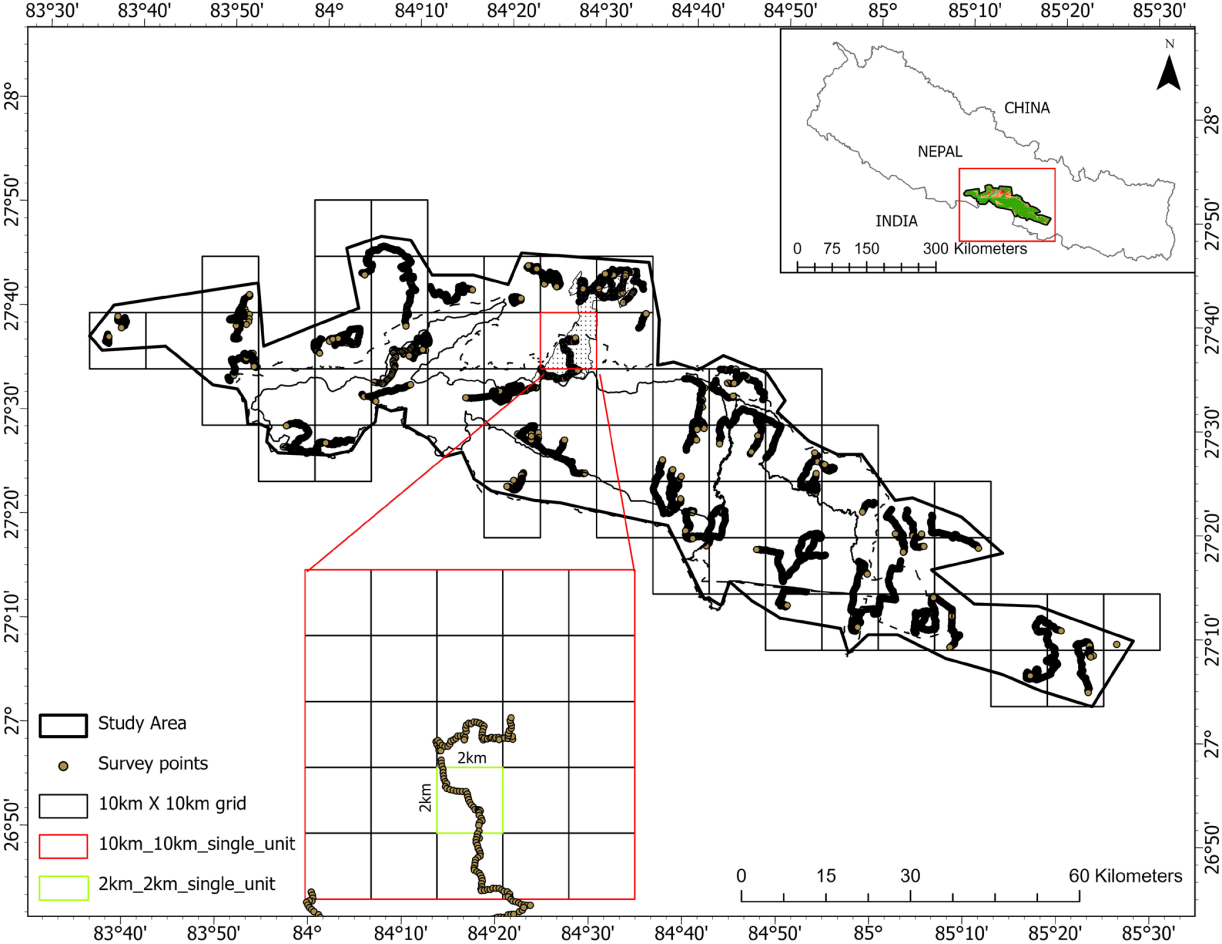
The study area was divided into 10 km × 10 km grids, which were further subdivided into 2 km × 2 km subgrids. Within each subgrid, data on the presence or absence of tigers and their prey species were collected at 100‐m intervals.

The study area was partitioned into 62 grids, each spanning 10 km by 10 km (Figure 2). The selection of this grid size was influenced by the knowledge that it exceeded the typical home range size of tigers in Chitwan National Park, Nepal. In this region, female tigers have home ranges of 16–39 km^2^, while males range between 60 and 105 km^2^ (M. E. Sunquist 1981; McDougal 1977). We surveyed all natural habitats within the landscape, including forests and prey‐rich grasslands (Chanchani et al. 2024), but excluded agricultural fields and densely human‐dominated areas. Each grid was further divided into subgrids of 2 km × 2 km each (*n* = 1550) for spatial replication. Each survey team, comprising 4–5 members—including university students, a local individual with wildlife knowledge, and a park staff—was led by experienced wildlife technicians (with over 10 years of experience) and officers from the respective park and the National Trust for Nature Conservation. All team members received refresher training from tiger experts prior to the survey. Within every grid, the survey teams traveled along a continuous random transect covering a maximum distance of 40 km, recording the presence of focal species every 100 m. Due to challenging terrain and logistical constraints, not all subgrids could be entirely surveyed, resulting in variations in survey efforts among the grids. The survey team prioritized the exploration of existing trails and unpaved roads wherever feasible to reduce the likelihood of incorrect absence records. This comprehensive survey was carried out between December 2021 and February 2022 by the Department of National Parks and Wildlife Conservation (DNPWC), Nepal.

In each surveyed subgrid, data was systematically collected to ascertain the presence of tigers and various prey species. This involved noting the existence or absence of tracks, fresh droppings, signs of feeding, territorial markings, and other relevant indicators (Lamichhane et al. 2021). Distinctions between tiger and leopard tracks were made using size‐based criteria, including pad size width (tiger not < 7 cm, leopard not > 6.4 cm), front foot width (tiger: 9–12 cm, leopard: ~7 cm), adult stride length (tiger: > 100 cm, leopard: ~90 cm), and claw‐scraping (tiger: > 35 cm height and > 19 cm width; leopard: < 25 cm height and < 15 cm width) measurements and other parameters (Simcharoen et al. 2018; Kolipaka 2007; Smith et al. 1989; Kafley et al. 2019; Jhala et al. 2011). Moreover, prey species were identified by analyzing their pellets, track shapes, and sizes. The specific measurements for sambar (*Rusa unicolar*), spotted deer ( *Axis axis* ), barking deer (*Muntiacus vaginalis*), wild boar ( *Sus scrofa* ), gaur ( *Bos gaurus* ), blue bull ( *Boselaphus tragocamelus* ) were referenced from Menon and Daniel (2009) and Kolipaka (2007) to ensure accurate identification. There were five 10 × 10 km grids which were unable to be visited by survey teams. In these grids, the presence of prey species was unknown, thus for these five cells we imputed missing prey values by averaging the observed prey in adjacent cells.

### 2.3 Variables Influencing Occupancy and Detection

We include several covariates to explain occupancy and detection during the sign survey: the normalized difference vegetation index (NDVI), human population density (HPD), and elevation (Elev), along with survey observations of prey and predators at 100 m intervals. Average NDVI, HPD and Elevation for 2 × 2 and 10 × 10 km grids were evaluated by taking spatially weighted grid means using the *exactextractr* library (Baston 2022). NDVI indices based on 250‐m resolution MODIS satellite imagery were obtained for February 2023 (Didan et al. 2015), and the 2000 Shuttle Radar Topology Mission 90‐m DEM (Jarvis et al. 2008) were used to evaluate the average values within each 2 × 2 km and 10 × 10 km grid cell. Elevations and NDVI were centered and scaled prior to modeling. Ward‐level census data for the year 2021 was obtained from the National Statistics Office (NSO) for Bagmati, Madhesh, Lumbini, and Gandaki provinces and matched to ward polygons for administrative regions of Nepal to derive an accurate map of human population density. Municipal wards are the finest spatial scale at which human population is reported by the NSO and are collected every 10 years (Table 1). After calculating the population density for all wards, we intersected wards with major park boundaries and assigned regions inside the Chitwan and Parsa park cores and the Barandabar corridor forest zero population. The resulting population map was used to evaluate the average population density within each survey grid and subgrid. Subgrids which obtained zero density were set to the minimum nonzero population density observed (11 per km^2^) before transforming both grid and subgrid populations to a $\log_{10}$ scale for modeling. Sampling effort by grid was calculated as the number of kilometers of trail surveyed and included in the detection model as a covariate. Prior to analysis, all covariates were standardized and compared for correlation. The correlation (Pearson) between standardized Prey, NDVI, HPD and Elev were below 0.60 at both the grid and subgrid scale (Table 1). A moderately strong correlation existed between standardized NDVI and HPD of −0.58, however we decided to retain all the variates in the model (Table 2, Figure S3).

**TABLE 1:** Definition and predicted effect of covariates in detectability and occupancy of tigers in the study area. Covariates were created at the 2 × 2 km subgrid scales and subgrids were averaged to produce 10 × 10 km grid scale covariates.

Covariates	Definition	Expected effect
Prey observed	The km of surveyed trails containing prey species (Sambar, Chital, Wild boar, Barking deer, Gaur, Blue bull) across the continuous random transects falling within a subgrid.	Positive (Ψ, known to be the function of carnivore densities, Karanth et al. 2011)
Elevation	Elevation averaged within subgrid cells, based on 90 m SRTM DEM (Jarvis et al. 2008).	Negative for Ψ and *p* (higher elevation are often associated with rugged terrain regions and tend to be difficult to access and less favored by tigers Lamichhane et al. 2025)
Normalized Difference Vegetation Index (NDVI)	NDVI averaged within subgrid cells, calculated from 250‐m MODIS satellite imagery for February 2023 (Didan et al. 2015).	Positive for Ψ (provides an opportunity for tiger, ambush predator) to hunt (Sharma et al. 2015), negative “*p*” (our study period was post‐monsoon, and the leaves shedding from the deciduous tree during this season reduces the chances of sign detection)
Human Population Density	Ward population divided by ward area, averaged within subgrid cells.	Negative for Ψ (increased population density within a grid increases human disturbance)

**TABLE 2:** Pearson correlation coefficient (*r*) between the standardized environmental covariates in the study area, within 10 × 10 grids and 2 × 2 km subgrids.

Grid	Prey	NDVI	HPD	Subgrid	Prey	NDVI	HPD
NDVI	0.1671			NDVI	0.1914		
HPD	−0.4456	−0.5795		HPD	−0.3576	−0.3526	
Elev	−0.4515	0.3684	0.0070	Elev	−0.1857	0.3183	0.0017

### 2.4 Occupancy Modeling

We constructed a Bayesian occupancy model for tigers in the CPC and surroundings using the JAGS library (Plummer 2017) in R. Our model considers two levels of occupancy for tigers: occupancy at the 10 km × 10 km grid scale and a finer division at the 2 km × 2 km subgrid scale, which we refer to as “use”. Prey use of subgrids was also modeled to better enable estimation of tiger subgrid use within occupied grids.

The occupancy of tigers in the $i^{\mathrm{th}}$ large grid $Z_{i},$ was allowed to vary logistically with NDVI, HPD Elevation and prey occupancy as measured at the large grid scale. Tiger use of subgrid *j* within grid *i*, $z_{\mathrm{ij}}^{y},$ was permitted to depend logistically on modeled prey subgrid use, $z_{\mathrm{ij}}^{x},$ as well as subgrid NDVI, HPD and Elevation, whereas prey subgrid use only depended on subgrid NDVI, HPD and Elevation. Total detections of tigers, $y_{\mathrm{ij}},$ and prey, $x_{\mathrm{ij}},$ during sign surveys were modeled as binomial within subgrids, with a maximum number of observations depending on subgrid survey effort $n_{\mathrm{ij}}$. Uniform priors were used for coefficients of both the occupancy and detection models.

The model is expressed as follows:

Grid occupancy:

$$Z_{i}∼\text{Bernoulli}\left(\psi_{i}\right)$$

$$\log\frac{\psi_{i}}{1-\psi_{i}}=b_{0}+b_{1}\cdot {\text{Prey}}_{i}+b_{2}\cdot {\text{NDVI}}_{i}+b_{3}\cdot {\mathrm{HPD}}_{i}+b_{4}\cdot {\text{Elev}}_{i}$$

Subgrid occupancy (use):

$$z_{\mathrm{ij}}^{x}∼\text{Bernoulli}\left(\psi_{\mathrm{ij}}^{x}\right)$$

$$z_{\mathrm{ij}}^{y}∼\text{Bernoulli}\left(\psi_{\mathrm{ij}}^{y}\right)$$

$$\log\frac{\psi_{\mathrm{ij}}^{x}}{1-\psi_{\mathrm{ij}}^{x}}=\beta_{0}^{x}+\beta_{2}^{x}\cdot {\text{NDVI}}_{\mathrm{ij}}+\beta_{3}^{x}\cdot {\mathrm{HPD}}_{\mathrm{ij}}+\beta_{4}^{x}\cdot {\text{Elev}}_{\mathrm{ij}}$$

$$\log\frac{\psi_{\mathrm{ij}}^{y}}{1-\psi_{\mathrm{ij}}^{y}}=\beta_{0}^{y}+\beta_{1}^{y}\cdot z_{\mathrm{ij}}^{x}+\beta_{2}^{y}\cdot {\text{NDVI}}_{\mathrm{ij}}+\beta_{3}^{y}\cdot {\mathrm{HPD}}_{\mathrm{ij}}+\beta_{4}^{y}\cdot {\text{Elev}}_{\mathrm{ij}}$$

Detection:

$$\log\frac{p_{\mathrm{ij}}^{x}}{1-p_{\mathrm{ij}}^{x}}=d_{0}^{x}+d_{1}^{x}\cdot {\text{NDVI}}_{\mathrm{ij}}+d_{2}^{x}\cdot {\text{Elev}}_{\mathrm{ij}}$$

$$\log\frac{p_{\mathrm{ij}}^{y}}{1-p_{\mathrm{ij}}^{y}}=d_{0}^{y}+d_{1}^{y}\cdot {\text{NDVI}}_{\mathrm{ij}}+d_{2}^{y}\cdot {\text{Elev}}_{\mathrm{ij}}$$

$$x_{\mathrm{ij}}∼\text{Binomial}\left(p_{\mathrm{ij}}\cdot Z_{i}\cdot z_{\mathrm{ij}}^{x},n_{\mathrm{ij}}\right)$$

$$y_{\mathrm{ij}}∼\text{Binomial}\left(p_{\mathrm{ij}}\cdot z_{\mathrm{ij}}^{y},n_{\mathrm{ij}}\right)$$

We simulated a realistic test dataset to verify the model's ability to recover the desired parameters. Covariates were generated from standard normal distributions and combined with plausible coefficients to generate occupancy status and detection rates as increasing logistically with realistic assigned coefficient values. The simulated survey effort was sampled from a distribution based on the observed survey effort frequency to ensure the number of visits per subgrid would be realistic. The above model fits JAGS using 10,000 Monte Carlo Markov Chain (MCMC) iterations with three chains, a thinning rate of 10, and a burn in rate of 2500. We observed the model successfully converged (R‐hat value < 1.1, Gelman and Hill 2007) and were able to recover the simulated parameters (Figure S1) successfully, so we proceeded to apply the model to the actual observed data.

### 2.5 Tiger Rescues and Rescue Data Analysis

The DNPWC leads the rescue decision‐making and site selection for the release of all problem tigers within the CPC. A rescue operation is initiated after a single human attack or at least three livestock‐killing incidents (Box 1). The tigers captured during such conflict events are usually individuals that are newly dispersed subadults, tigers involved in territorial conflicts, or aged tigers. During the rescue, teams evaluate each tiger's age and overall health condition, including dentition. If experts assess that the tiger has a high likelihood of survival, it is released into a core area far from buffer zones and human settlements, preferably in sites with continuous camera monitoring, regular army patrols, and no known conflict‐prone tigers. If the team determines that the tiger's survival prospects are low, it is transferred to an enclosure for care and management. Whenever possible, releases are carried out within the same protected area, or, if the tiger is captured in a divisional forest, in the nearest suitable protected area (Dr. Ashok Kumar Ram, Chief Warden, Bardia National Park, Personal Communication).

BOX 1 **Rescue response to a human‐tiger conflict in Nepal, showing post‐immobilization examination of an adult male tiger in the Chitwan‐Parsa complex.**

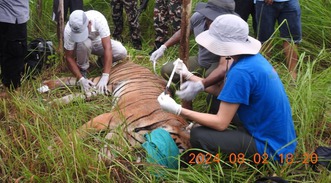

Upon receiving a report of an injured, abandoned, or conflict tiger, the Department of National Parks and Wildlife Conservation (DNPWC), with technical assistance from NTNC field offices, launches a rescue operation. First, the conflict site is secured with crowd and traffic control, and in parallel, camera traps are deployed around the incident area to confirm the tiger's presence; once images are obtained, the individual is cross‐checked against the park's photo ID archive from recent surveys (e.g., 2018, 2022) to verify identity, sex, age class, and recent history, which guides the intervention plan. A rapid risk assessment is conducted to determine the safest intervention. Essential equipment, including personal protective gear, veterinary immobilization tools, a stretcher, a secure transport enclosure, and monitoring devices, is prepared, and team roles and communication are clearly assigned. If chemical immobilization is required, the veterinarian will administer anesthesia from a safe position, after which the tiger's airway, vital signs, and temperature are monitored, stress minimized, urgent injuries treated, and minimal authorized data collected. The rescued tiger is then placed in a covered, ventilated enclosure, secured to prevent injury, and transported along the safest, most direct route under continuous monitoring. At the approved site, anesthesia is reversed and released only when the tiger is stable and the area is secure. Finally, the incident is recorded, the team will review the operation, and the local community is informed to help prevent future conflicts. Photo: Rescue teams examine an adult male tiger involved in a conflict in the south‐central CPC buffer region east of Thori. The subgrid in which this rescue operation was conducted had below average population density and above average prey use. (Photo credit: NTNC—BCC).

With the permission of DNPWC and NTNC, we obtained records of tiger rescues during the period January 2011–August 2024. We selected only tiger rescues from within the vicinity of the CPC dating back to the start of year 2020 for analysis. Following the conclusion of occupancy modeling, we constructed contingency tables to assess associations between subgrid use of tigers, prey, and human population with observed tiger rescue sites within the CPC buffer region. Within the CPC buffer region, we classified 2 × 2 km^2^ subgrids as high and low use by tiger and prey use based on whether they fall above or below the mean use rate across the buffer zones. High human population subgrids exceeded the average log population (141 persons per km^2^). Tests for association were conducted using log odds ratios, and for common odds ratios across tables using a chi‐square test (van Belle et al. 2004).

## 3 Results

The survey team covered a total of 808.5 km to document tiger indications and prey occurrence. Across the 57 surveyed grids (10 km by 10 km), a total of 184 tiger signs were detected across 2 km by 2 km subgrids in 36 grids, yielding a naïve tiger occupancy of 0.63. Barking deer were the most frequently observed prey species, with sightings in 46 grids (80%). Other prey species, including sambar, chital, and wild boar, were observed in 38, 13, and 13 grids, respectively.

**FIGURE 3:**
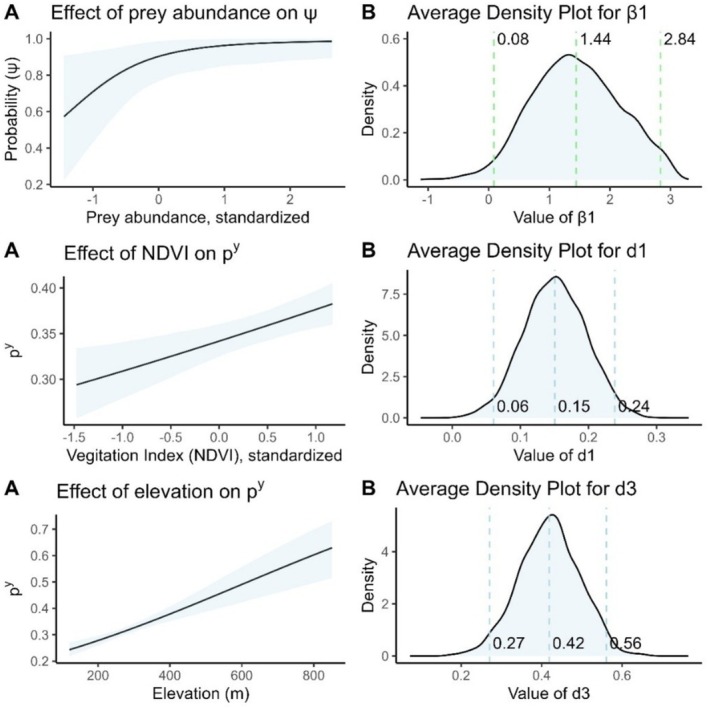
Top panel: Relationship between probability of tiger occupancy (Ψ) and prey abundance, in standard deviations. Middle and lower panels: Relationships between tiger detection probability (*p* ^*y*^) and normalized difference vegetation index (NDVI) and elevation. Panels labeled A illustrate the estimated probability and credible interval with covariates not depicted evaluated at their mean over all grids, and panels marked B show the distribution of the associated parameter estimates, with mean and 95% credible interval for the detection coefficients.

The tiger occupancy of 10 km × 10 km grids at the mean covariate values for grids was estimated to be 0.903 (CI 0.768–98.6), whereas the use rate for 2 km × 2 km subgrids at the average covariate levels was 0.462 (CI 0.288–0.651). At the grid scale, tiger occupancy was positively influenced by the prey abundance ($b_{1}=1.42,\;\mathrm{CI}:0.08,2.84$) (Figure 3). Tiger occupancy was estimated to be negatively affected by NDVI ($b_{2}=-0.852$, CI: −2.74, 1.37), HPD ($b_{3}$ = −0.841, CI: −1.546, 0.0675), and Elevation ($b_{4}$ = −0.784, CI: −2.566, 0.940), but the evidence was not strong enough to conclude that the coefficients were nonzero at the grid scale (Figure S2).

The mean detection rate for tigers and prey in subgrids with the mean covariate values of subgrid NDVI and Elevation was estimated to be 0.342 (CI: 0.325, 0.360) and 0.62 (CI: 0.61–0.66), respectively. Tiger detection was positively affected by NDVI (*d*_1_ = 0.15, CI: 0.06–0.24) and Elevation (*d*_3_ = 0.42, CI: 0.27–0.56) (Figure 3). Similarly, prey detection was positively affected by NDVI (*c*_1_ = 0.43, CI: 0.36–0.49) and negatively affected by Elevation (*c*_3_ = −0.65, CI: −0.73 to −0.58) (Figures S3 and S5).

Tiger use of subgrids was significantly positively related to NDVI ($\beta_{2}^{y}$ = 0.583, CI: 0.225, 0.956), and negatively with HPD ($\beta_{3}^{y}$ = −0.664, CI: −0.873, −0.459) and Elevation ($\beta_{4}^{y}$ = −1.264, CI: −1.789, −0.763). Subgrid use was positively related to prey subgrid use ($\beta_{1}^{x}$ = 0.570, CI: −0.352, 1.445), but the relationship was not strong enough to conclude prey use subgrid use positively affected tiger subgrid use. Prey subgrid use was significantly negatively related to HPD and Elevation, but not to NDVI (Table S1, Figures S4 and S5).

**FIGURE 4:**
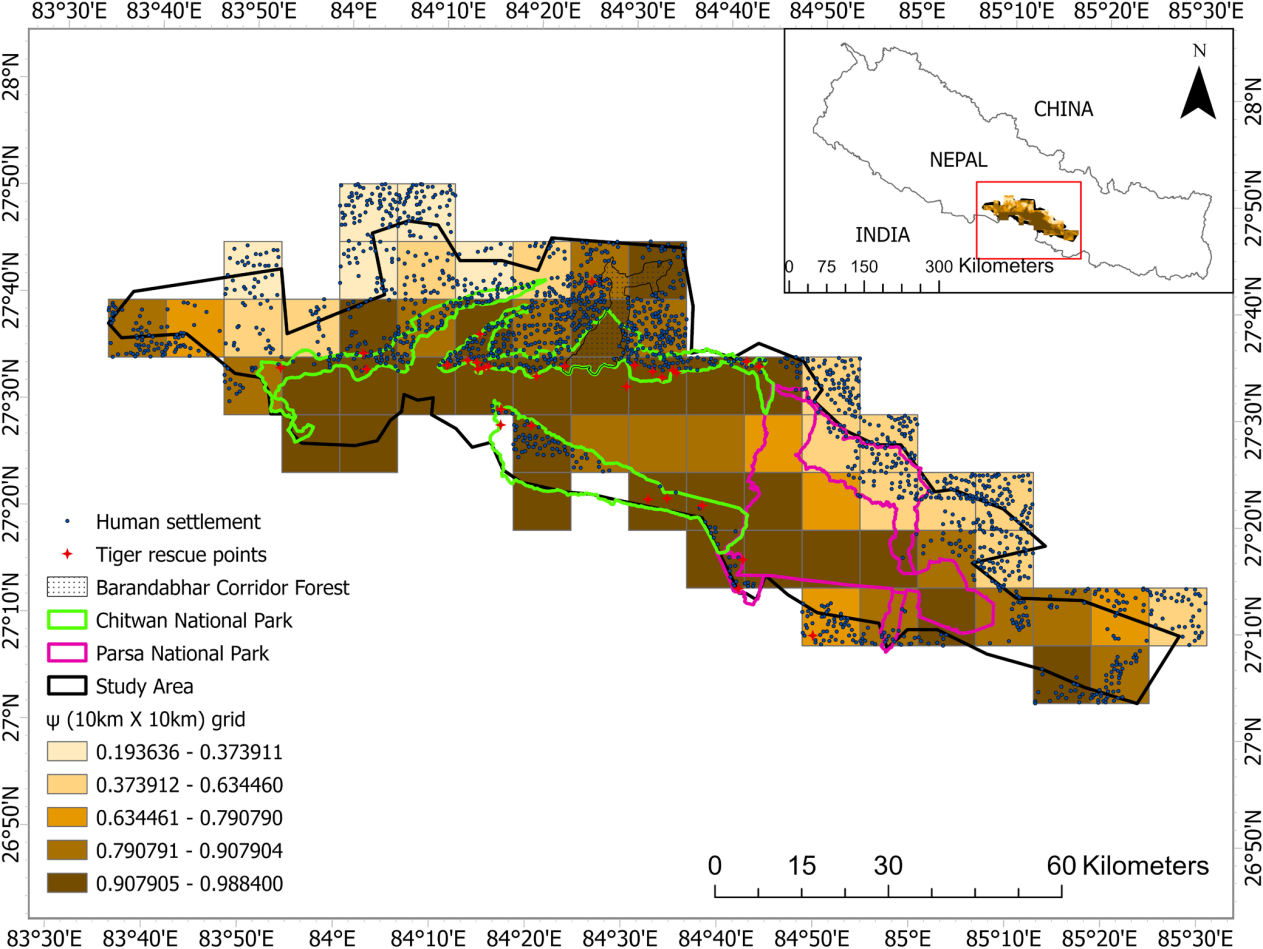
Probability of site occupancy (ψ) of tigers in the study area. The study area covers the Chitwan–Parsa Complex and the forested area adjoining the human settlements. The human settlements are shown on the map. The red star shows the five years tiger rescued points (2020–2024). “BZ” refers to the Buffer zone of the respective protected areas.

**FIGURE 5:**
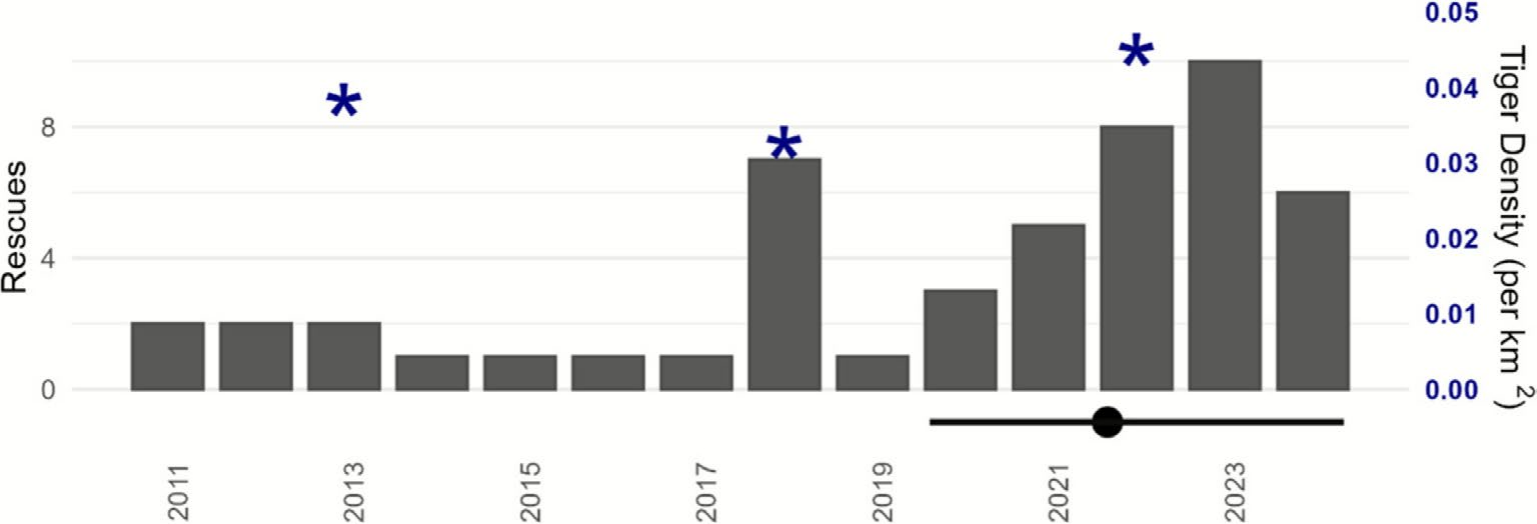
Timeline of tiger rescues in the vicinity of the CPC between January 2011 and August 2024. Blue stars (*) indicates population density of tigers in Chitwan estimated during the three most recent national tiger surveys in 2013, 2018 and 2022. The solid black horizontal line indicates the rescue years used to examine the recent pattern of tiger rescues, for comparison against the modeled spatial distribution of tiger occupancy during winter 2021–22 (solid black dot).

Veterinary teams relocated a total of 32 tigers in the vicinity of Chitwan and Parsa national parks between May 2020 and August 2024 (Figures 4 and 5; Box 1). Rescued tigers were predominantly adult males (17); seven adult females were rescued, and the remainder were subadults (1 female) or cubs (2 female, 1 male). The age of four male tigers was not reported (Table 3). We considered whether rescues were affected by prey use, tiger use and human population individually by comparing the odds of rescues at levels above and below the mean of each variable of interest within the 2 × 2 km buffer subgrid cells (Table 3) with a one‐sided *Z*‐test for the log‐odds ratio (van Belle et al. 2004). The odds of tiger rescues in the buffer areas in which humans had above average populations were 1.10 times higher than the odds of rescue in low human population buffer subgrids (log odds 0.0904, *Z* = 0.172, *p* = 0.432). Areas with above average human populations had odds of rescues 2.2 times those of low population areas (log odds 0.773, *Z* = 1.409, *p* = 0.079). However, the odds of tiger rescues within subgrids with high prey use were 3.8 times higher than those with below average prey use (log odds 1.338, *Z* = 2.064, *p* = 0.020), which provides strong evidence that above average prey subgrid use is associated with increased odds of rescue in the buffer zone.

**TABLE 3:** Two‐way contingency tables comparing the number 2 × 2 km subgrids with and without tiger rescues among sites with above or below average tiger use ($\psi_{\mathrm{ij}}^{y}>0.54$), log population density (> 141 persons/km^2^), and prey use ($\psi_{\mathrm{ij}}^{x}>0.80$).

		Tiger use		Population density		Prey use	
		High	Low	High	Low	High	Low
Tiger rescue	Yes	11	5	10	6	13	3
	No	197	194	236	155	208	183

To examine whether the relationship between rescues and prey use varies with human population density, we separate the dataset into the buffer subgrids with human populations above (*n* = 246) or below (*n* = 161) the average human population density of 141 persons per km^2^ and apply a test for homogeneity of odds ratios (Figure 5; Tables 3 and 4). Under the null hypothesis of no interaction, the odds ratio should be similar across each table, which can be tested through a chi‐squared test (van Belle et al. 2004). In sites with above average population density, the odds of rescues in high‐prey areas were 10.98 times higher than the odds of rescues in low‐prey areas (Table 4). This contrasted with low prey‐regions, where the odds of tiger rescues occurring were 2.9 times higher in subgrids with low‐prey than in those with high‐prey (Tables 3 and 4). Together this provides strong evidence that the odds ratios were not constant across tables ($\chi_{2}=7.785$, *p* = 0.0204).

**TABLE 4:** Three‐way contingency table for examining interactions between the rates of rescues in 2 × 2 km subgrids with varying levels of prey use and population density. High population density sites exceed 141 persons per km^2^ and high prey density sites exceed use rates of $\psi_{\mathrm{ij}}^{x}>0.80$.

		Low pop. density		High pop. density	
		Prey use		Prey use	
		High	Low	High	Low
Tiger rescue	Yes	5	1	8	2
	No	145	10	63	173

**FIGURE 6:**
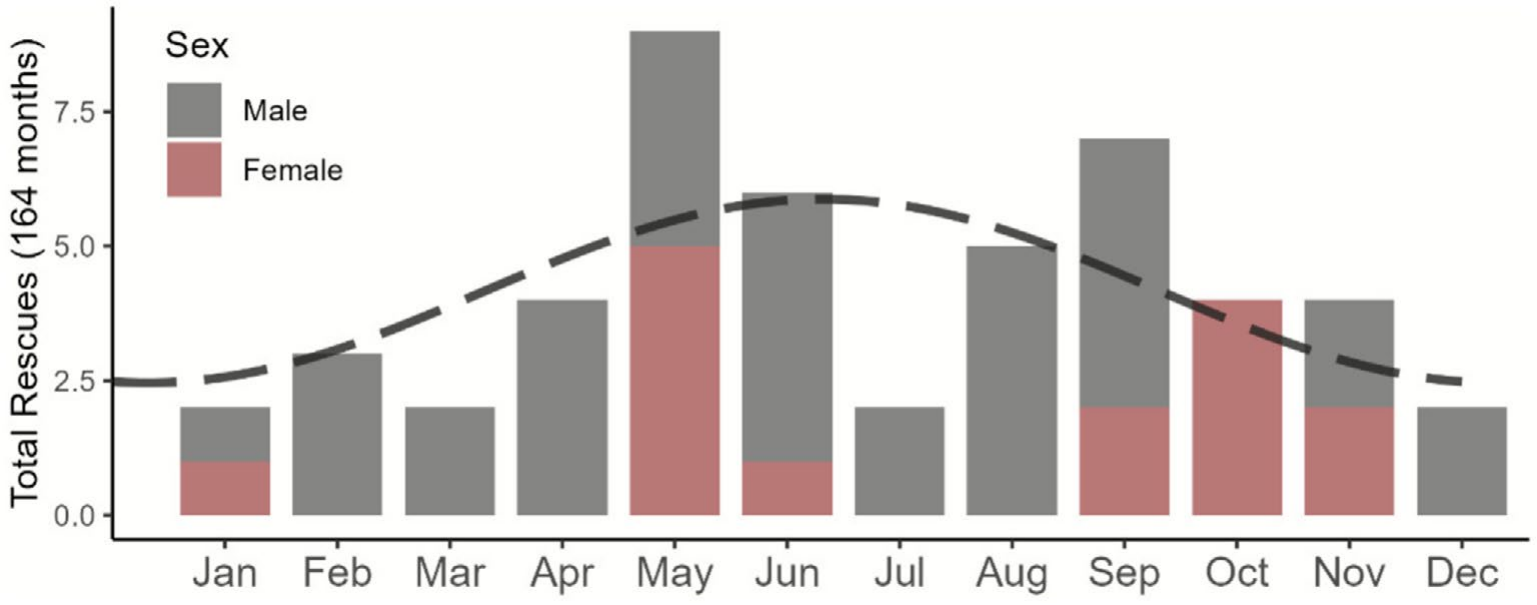
Seasonal pattern of tiger rescues within the Chitwan–Parsa Complex and adjoining forested areas between January 2011 and August 2024. The dashed line overlaid represents the best fitting sinusoidal curve based on squared error loss.

Tiger rescues within the CPC buffers showed seasonality when examined over the 164 months period going back to 2011 (Figures 5 and 6). We approximated the shape of the distribution by fitting a sinusoid and minimizing the mean square error for the total number of rescues by month over the period. The expected maximum rate of 5.9 total rescues over 14 years occurred at the start of July and expected minimum of 2.5 over 14 years occurred at the end of January (Figures 5 and 6; Table 5). This calendar aligns with tiger reproductive phenology in the subcontinent: breeding occurs year‐round but peaks from roughly November to April, with a gestation of about 3.5 months (Figure 6).

**TABLE 5:** Age and sex of tigers within the CPC and buffers relocated by veterinary teams between January 2020 and August 2024.

	Adult	Cub	Subadult	Unknown
Female	7	2	0	0
Male	17	1	1	4

*Note:* Data on the age of four tigers was not available.

## 4 Discussion

The recovery of tigers in the Chitwan–Parsa Complex (CPC) is a conservation success, but it also expands interfaces with people. Using a two‐scale occupancy framework (10 × 10 km grids; 2 × 2 km subgrids), we estimated high grid‐level occupancy (Ψ ~ 0.90) and found that grid occupancy increased with prey abundance, while subgrid use rose with vegetation cover (NDVI) and declined with human population density and elevation; detection increased with habitat (NDVI, elevation). Coupling these patterns with 2020–2024 rescue records shows rescues concentrate in prey‐rich buffer subgrids, and where human densities are higher, the odds of rescue in high‐prey areas are markedly elevated, underscoring the need to prioritize conflict mitigation and proactive monitoring at prey‐rich, high‐density buffer interfaces, and to refine release‐site decisions to reduce repeat conflict while sustaining conservation gains.

Our results show that tiger occupancy in some buffer grids was higher than expected near human settlements, such as those in Thori and Madi municipalities, with increased tiger rescues reflecting a greater risk of human–tiger conflict. In Chitwan National Park (CNP), conflict‐related tiger rescues were particularly high (Figure 4), corresponding with the rise in tiger populations within the CPC from 127 individuals in 2010 to 169 in 2022 (DNPWC and DFSC 2022). In CNP alone, tiger numbers grew from 91 in 2009 to 128 in 2022, and human fatalities from tiger conflicts reached 13 in 2022, compared to no record in 2010 (CNP 2010, 2022). Gurung et al. (2008) found that most human fatalities in CNP involved fodder collectors. Further, the 2022 tiger survey recorded 15 tigers in forested areas outside the CPC, up from just four in 2018 (DNPWC and DFSC 2018), with some tigers even camera‐trapped in the remote Chure region (Subedi et al. 2021). These findings highlight the need for caution during fodder collection or livestock grazing in areas with high tiger occupancy and frequent rescues. At the same time, conservation managers should prioritize interventions in these zones to minimize human casualties.

The results indicate a negative effect of human population density (HPD) on tiger occupancy; however, the evidence as a significant predictor is limited, as the credible interval (CI) overlaps with zero, preventing rejection of the null model at *α* = 0.05. This suggests that while HPD may influence occupancy, its effect appears weak or inconsistent within the measured areas, possibly due to unaccounted factors modulating its relationship with occupancy (ψ). For example, although we used HPD at the grid level, a more precise measure might be the number of people visiting forested areas, as not all residents living near forests enter them. Incorporating such data could offer deeper insights into the relationship between human activity and tiger occupancy. Notably, HPD significantly negatively affected tiger and prey habitat use, indicating that prey and predators avoid areas with higher human density at a fine scale (Figure S4).

While NDVI was expected to influence tiger occupancy probability positively, our results show no significant effect, possibly reflecting biases in survey design, as monitoring efforts outside protected areas were concentrated in forested regions with higher conflict frequency. However, though not statistically significant, the observed positive association between NDVI and tiger occupancy suggests that tigers prefer forested habitats, reinforcing the link between habitat quality and tiger distribution. This pattern also aligns with human–tiger interactions, as livestock grazing and fodder collection predominantly occur in forested areas outside the protected areas, increasing the likelihood of livestock depredation and human loss. For example, 61 livestock depredation cases from tigers were reported across the buffer zone of CNP in 2022 (CNP 2022). These findings underscore the context‐dependent nature of occupancy patterns, highlighting the need for more nuanced management strategies in areas with high tiger occupancy to mitigate conflict risks effectively.

The prey showed a positive effect on tiger occupancy. Tigers, being large predators, rely on a substantial prey base and often avoid livestock when wild prey abundance is ample (Biswas and Sankar 2002; Reddy et al. 2004). Previous studies on the tiger diet did not reveal evidence of livestock consumption within the Chitwan and Parsa National Parks (Lamichhane and Jha 2015; Pun et al. 2022). Furthermore, the prey density per km^2^ in these protected areas substantially sustains the present tiger number (Lamichhane et al. 2024). However, the prey density is not evenly distributed in the park (DNPWC and DFSC 2022), enhancing the likelihood of livestock depredation, as seen above by the number of predation cases. The observed positive and significant impact of the wild ungulate on tiger occupancy underscores the critical importance of maintaining adequate prey populations in the study area. Furthermore, the practice of corralling livestock outside the parks during the night and predator‐proof livestock enclosure contributes to a reduction in the likelihood of predation (Gurung et al. 2010; Kolipaka et al. 2017).

Our findings indicate that tiger detection rates are higher at elevated locations. In high‐elevation areas, the movement of tigers, being solitary hunters, is restricted due to the limited availability of tracks or trails, often with only a single trail present (Hines et al. 2010). This constraint increases the likelihood of detecting tiger signs. Similarly, for NDVI, tigers prefer areas with cover suitable for ambush hunting, which may explain the increased detection probability in such habitats. The detection probability of prey was positively influenced by NDVI, suggesting their preference for areas with higher vegetation cover, which improves their detectability. However, at higher elevations, detection probability decreases, likely due to the limited availability of tracks or trails, often reduced to just a single path in some cases. These conditions may increase the encounter rate with predators or other disturbances, prompting prey species to avoid such trails to minimize risks. This avoidance behavior likely explains the reduced detection probability of prey at higher elevations.

Across the buffer zones around Chitwan and Parsa, 32 tigers were relocated between the start of 2020 and August 2024, with rescues predominantly adult males (Table 5; Box 1). This may be because males are the primary dispersing sex and have a larger territory than females. Overall, use by tigers on its own did not necessarily lead to higher odds of rescues within a region (OR ≈ 1.10, *p* = 0.432); however, there was a significant increase in odds of rescues in the presence of elevated prey use (OR ≈ 3.8, *p* = 0.020). Furthermore, this elevated risk of rescues in the presence of prey primarily arises within subgrids with elevated human population (OR ≈ 10.98, *p* = 0.0014).

By and large, prey tends to prefer buffer subgrids with lower human population density; for instance, there was a negative Pearson correlation between subgrid prey and human population density. From an odds perspective, the odds of above average prey use were 33.6 times higher in buffer subgrids with below average human population than in those with above average human population. However, the increase in odds of rescues in the presence of humans and prey was substantial enough to result in most rescues (14 out of 20) occurring in high‐prey, high population subgrids (Table 5). Among rescues that occur in below‐average human population density regions, the majority occur within high prey regions; however, this is due to the overwhelming number of low‐population subgrids which have above average prey. There is suggestive evidence that the rescue rate in low‐population, low prey regions may be non‐negligible: Of the eleven low population, low prey subgrids in the buffer, a rescue was observed in one case, that of an adult male tiger captured just north of Chitwan, on the north bank of the Narayani. The region to the north is largely agricultural; however, there are isolated patches of trees near the water which could have lured a tiger from Chitwan but been insufficient in size to support the tiger on wild prey alone. It would also have been a hurdle for the tiger to cross the Narayani, forcing it to decide between approaching humans and crossing a large river—the width of the river at that point being nearly 200 m.

This pattern is consistent with two processes: near settlements, prey‐rich subgrids attract tigers and intensify human–tiger encounters requiring rescue; in low‐human landscapes, rescues may arise where prey scarcity pushes tigers into atypical or conflict‐prone spaces. Hence, prioritizing rapid response and proactive risk reduction in buffer subgrids with high prey availability and dense human populations, while enhancing prey recovery and movement corridors in low‐population areas, could help lower rescue demand and mitigate conflict across the study area.

Although abundant large prey helps confine tigers within the forested areas, it does not eliminate the possibility of problematic tigers on the periphery. Factors such as the age and health of individual tigers can influence their behavior, potentially escalating the risk of livestock predation or even attacking humans (Goodrich 2010). Therefore, park management authorities should remain vigilant and proactive in monitoring and management efforts to minimize the risk of conflicts. They may identify conflict‐causing tigers (e.g., using camera traps in the reported conflict areas and identifying tigers from their unique patterns) and devise possible ways to manage them (e.g., removing the conflict one from the area). In Nepal, the government's revenue‐sharing policy—allocating 30%–50% of protected area income to communities in buffer zones—has helped improve local tolerance toward wildlife (HMGN 1996). In addition, information on socioeconomic factors such as household dependence on livestock (Gurung et al. 2010), the effectiveness of insurance schemes and compensation programs (Ravenelle and Nyhus 2017), and land‐use dynamics should be integrated into spatial risk assessments. For example, prioritizing livestock insurance for predator‐proof corrals in high‐risk zones can reduce tiger depredation events (Bhattarai and Fischer 2014). Further, policies that promote land‐use planning sensitive to tiger habitats can mitigate conflicts (Goodrich 2010).

## 5 Conclusion

Using a two‐scale occupancy framework across the Chitwan–Parsa Complex, we found high grid‐level tiger occupancy (Ψ ~ 0.90), which increased with prey abundance. Subgrid use was positively associated with vegetation (NDVI) but declined with higher human population density and elevation; while the prey–use effect on tiger use was positive, it was not strongly supported. Detection probabilities varied with habitat: tiger detection increased with NDVI and elevation, whereas prey detection increased with NDVI but decreased with elevation. Combining modeled use patterns for winter 2021–22 with recent rescue data (2020–2024) revealed that rescues were disproportionately concentrated in prey‐rich buffer subgrids and areas with higher human densities, showing a distinct seasonal pattern (peaking in early July and declining in late January). Moreover, the relationship between rescues and prey use shifted with human density, suggesting spatially heterogeneous conflict risks across the landscape. We caution that regions with physically restricted connectivity adjacent to human settlements may have different risk patterns. Those which look attractive due to the presence of narrow corridors may be sufficient for access by predators but insufficient availability of prey, and restricted connectivity may raise the odds of conflict. Collectively, these findings emphasize the need to sustain prey populations while prioritizing proactive monitoring and conflict mitigation in prey‐rich, high‐human density buffer zones, and to guide DNPWC's rescue decisions, particularly regarding timing, release or transfer sites, and post‐release monitoring, to balance conservation objectives with human safety.

## Author Contributions

**Anil Prasai:** data curation (equal), formal analysis (equal), methodology (equal), project administration (equal), validation (equal), visualization (equal), writing – original draft (lead), writing – review and editing (equal). **Saneer Lamichhane:** data curation (equal), formal analysis (equal), investigation (equal), methodology (equal), validation (equal), visualization (equal), writing – original draft (equal), writing – review and editing (equal). **Abhinaya Pathak:** investigation (equal), methodology (equal), project administration (equal), validation (equal), writing – review and editing (equal). **Ajay Karki:** investigation (equal), methodology (equal), project administration (equal), writing – review and editing (equal). **Trishna Rayamajhi:** data curation (equal), visualization (equal), writing – original draft (equal), writing – review and editing (equal). **Jeffrey Mintz:** data curation (equal), methodology (equal), validation (equal), visualization (equal), writing – original draft (equal), writing – review and editing (equal). **Bhagawan Raj Dahal:** funding acquisition (equal), project administration (equal), writing – original draft (equal), writing – review and editing (equal). **Bed Kumar Dhakal:** funding acquisition (equal), methodology (equal), project administration (equal), supervision (equal), writing – original draft (equal), writing – review and editing (equal). **Chiranjibi Prasad Pokheral:** conceptualization (equal), funding acquisition (equal), investigation (equal), project administration (equal), supervision (lead), validation (equal), visualization (equal), writing – original draft (equal), writing – review and editing (equal). **Naresh Subedi:** conceptualization (equal), data curation (equal), funding acquisition (equal), investigation (equal), methodology (equal), project administration (equal), supervision (lead), validation (equal), visualization (equal), writing – original draft (equal), writing – review and editing (equal). **Bishnu Prasad Bhattarai:** data curation (equal), investigation (equal), supervision (lead), writing – original draft (equal), writing – review and editing (equal).

## Conflicts of Interest

The authors declare no conflicts of interest.

## Figures and Tables

**FIGURE A1:**
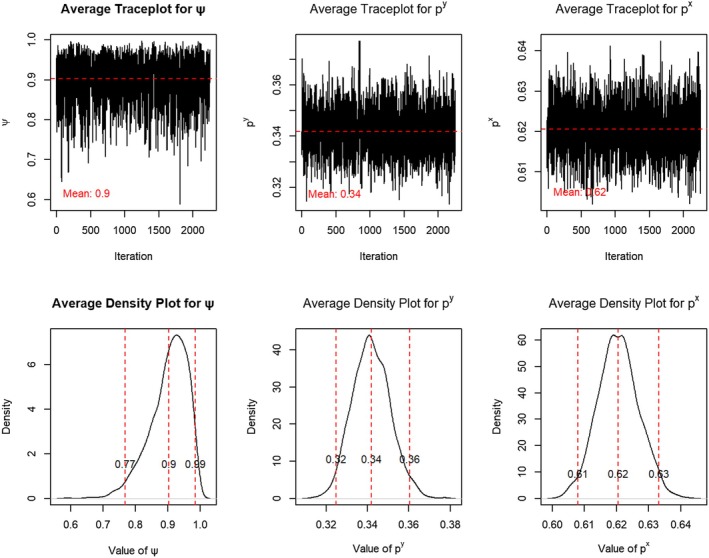
The traceplots (top) depict the Markov Chain Monte Carlo (MCMC) sampling of parameters for occupancy ψ, tiger detection $p^{y}$ and prey detection $p^{x}$, across 10,000 iterations. The *y*‐axis exhibits sampled values, while the *x*‐axis denotes the iteration number. The red dotted line represents the mean parameter value, which are overlaid in red. A burn‐in period of 2500 iterations was discarded to ensure model stabilization. Below each traceplot is a density plot for the posterior distribution of parameters ψ and $p^{x}$ and $p^{y}$ obtained from MCMC sampling, illustrating the distribution's shape, providing insights into parameter uncertainty. The curves are Gaussian kernel density estimates, offering a smoothed representation of the underlying distribution. The center vertical dotted line denotes the mean parameter value, and the outer vertical bars represent the limits of a 95% credible interval for the parameter.

**FIGURE A2:**
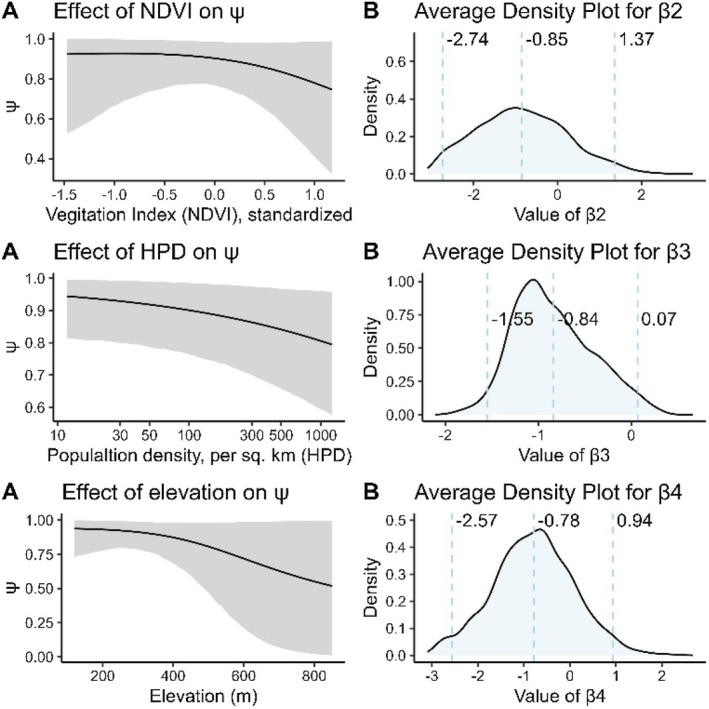
Relationships between non‐significant occupancy covariates and tiger occupancy in the study area. Panels labeled A illustrate the effect of each covariate on occupancy, and panels labeled B show the corresponding distribution estimated for the covariate on log odds of tiger occupancy. These three covariates were not found to significantly affect tiger occupancy, as the credible interval (CI) overlaps with zero in panel B. HPD, human population density; NDVI, normalized difference vegetation index (standardized).

**FIGURE A3:**
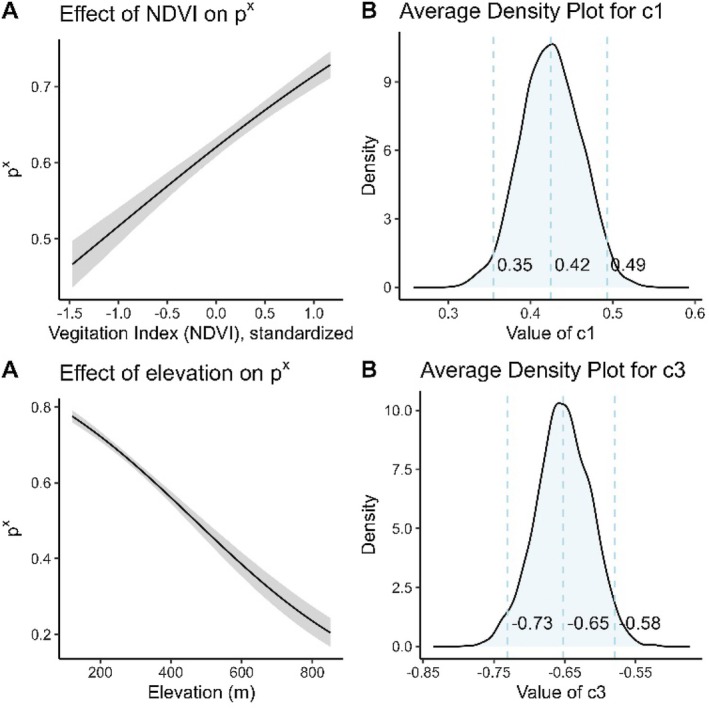
Relationship between normalized difference vegetation index (NDVI) and Elevation with prey detection probability (*p* ^*x*^). Panels labeled A show the effect of increasing values of the covariate on detection rates. Panels labeled B show the distributions estimated for the coefficients controlling the rates of change in detection with changes in NDVI or elevation, with overlaid median and 95% credible interval for the detection coefficients.

**FIGURE A4:**
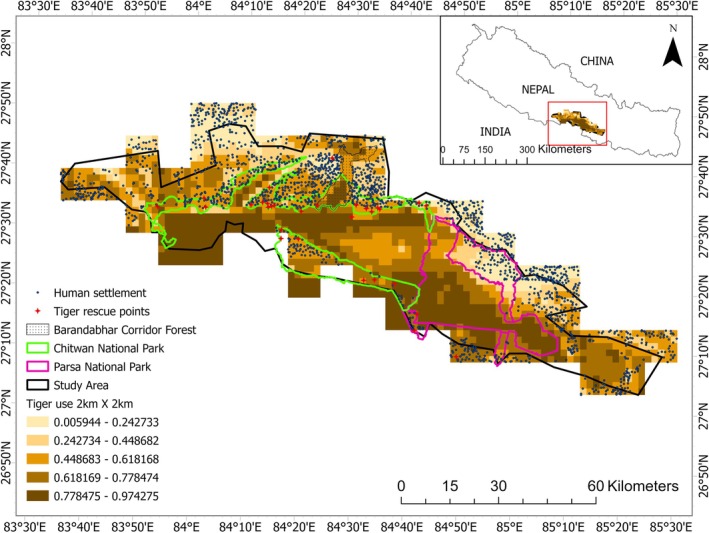
Probability of subgrid use of tigers in the study area. The study area covers Chitwan–Parsa Complex and the forested area adjoining the human settlements. The red star shows the five years tiger rescued points (2020–2024).

**FIGURE A5:**
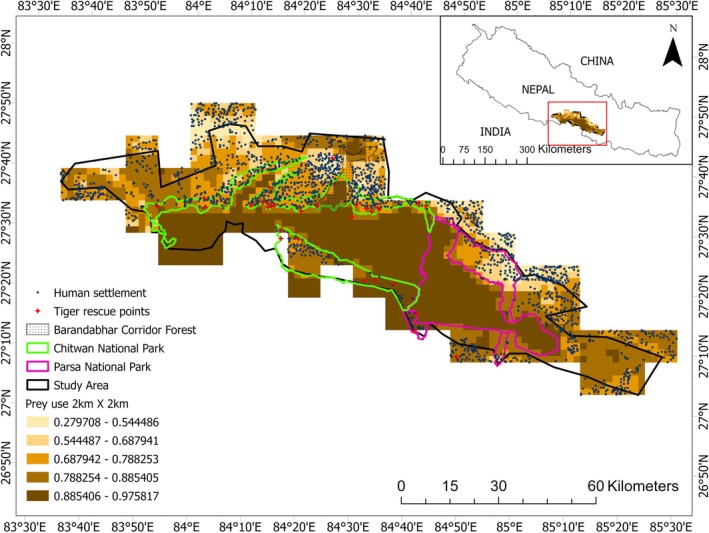
Probability of subgrid use of prey in the study area. The study area covers Chitwan–Parsa Complex and the forested area adjoining the human settlements. The red star shows the five years tiger rescued points (2020–2024).

## Data Availability

Code and Data are available at https://doi.org/10.5061/dryad.7h44j1057 (https://datadryad.org/dataset/doi:10.5061/dryad.7h44j1057). Additional information including data and results are provided in Appendix Information.

## Appendix 1

**TABLE A1:** Summary of model parameters showing estimates (q2.5, q50, q9.75), convergence diagnostics (R‐hat), and variable descriptions for predictors of tiger and prey use of habitat.

Parameter	q2.5	q50	q97.5	R‐hat	Variable
tiger0	−2.996	−2.879	−2.366	1.000	$\beta_{0}^{y}$
tiger_prey	−0.352	0.570	1.445	1.001	$\beta_{1}^{y}$
tiger_ndvi	0.225	0.583	0.956	1.001	$\beta_{2}^{y}$
tiger_hpd	−0.873	−0.664	−0.459	1.001	$\beta_{3}^{y}$
tiger_elev	−1.789	−1.264	−0.763	1.002	$\beta_{4}^{y}$
prey0	−2.987	−2.591	−1.402	1.002	$\beta_{0}^{x}$
prey_ndvi	−0.199	0.204	0.581	1.001	$\beta_{2}^{x}$
prey_hpd	−1.206	−1.061	−0.764	1.003	$\beta_{3}^{x}$
prey_elev	−0.930	−0.588	−0.263	0.999	$\beta_{4}^{x}$
deviance	5573.272	5596.390	5627.117	1.002	Model deviance

Abbreviations: Elev, elevation; HPD, human population density; NDVI, normalized difference vegetation index.
